# A protein- and fiber-rich diet with astaxanthin alleviates high-fat diet-induced obesity in beagles

**DOI:** 10.3389/fnut.2022.1019615

**Published:** 2022-10-24

**Authors:** Jinhua Xue, Yuanyuan Lu, Toujun Zou, Wei Shi, Shuang Wang, Xu Cheng, Juan Wan, Yun Chen, Min Wang, Qingzheng Wang, Xia Yang, Mingxing Ding, Zhili Qi, Yi Ding, Manli Hu, Xin Zhang, Hongliang Li, Yufeng Hu

**Affiliations:** ^1^Gannan Innovation and Transformation Medical Research Institute, Key Laboratory of Cardiovascular Disease Prevention and Control, Ministry of Education, First Affiliated Hospital of Gannan Medical University, Gannan Medical University, Ganzhou, China; ^2^Department of Physiology, School of Basic Medicine, Gannan Medical University, Ganzhou, China; ^3^Department of Cardiology, Renmin Hospital of Wuhan University, Wuhan, China; ^4^School of Basic Medicine, Wuhan University, Wuhan, China; ^5^College of Veterinary Medicine, Huazhong Agricultural University, Wuhan, China; ^6^Department of Cardiology, Huanggang Central Hospital, Huanggang, China; ^7^Jiangxi Huichong Technology Co., Ltd., Ganzhou, China

**Keywords:** obesity, canine, diet, weight loss, transcriptional profile

## Abstract

**Background and aims:**

Overweight or obesity is one of the most prevalent health burdens in companion pets and predisposes subjects to multiple comorbidities and reduced longevity. Dietary management and sufficient exercise are effective options for weight loss but challenged by modern lifestyle and calorie control-triggered malnutrition. Therefore, this study aimed to develop a formulated obesity control diet characterized by protein- and fiber-rich diet and supplemented with astaxanthin. We systemically evaluated global influences of the designed weight-loss diet on metabolic homeostasis in an obese beagle model.

**Materials and methods:**

Beagles were induced for obesity by a 24-week HFD treatment and then included into weight-loss programs. Briefly, obese beagles were randomly assigned to two groups that were fed with a formulated weight-loss diet or control diet, respectively. Body weight and body condition scoring (BCS) were analyzed biweekly. Computed tomography (CT), nuclear magnetic resonance imaging (MRI) measurements, and blood and adipose tissue biopsies were collected at 0 and 8 weeks. Plasma lipids and adipocyte size were also measured after 8 weeks of weight-loss diet feeding. The global influence of the formulated diet on the whole spectrum of gene panels were examined by adipose RNA assays.

**Results:**

Twenty-four weeks of continuous HFD feeding significantly induced obesity in beagles, as evidenced by increased body weight, BCS, abdominal fat mass, and serum lipid levels. The obese and metabolic condition of the modeled canine were effectively improved by an 8-week weight-loss diet administration. Importantly, we did not observe any side effects during the weight loss duration. Transcriptional analysis of adipose tissues further supported that a weight-loss diet significantly increased energy metabolism-related pathways and decreased lipid synthesis-related pathways.

**Conclusion:**

The prescribed weight-loss diet exhibited profound benefits in canine weight management with well safety and palatability. These findings support effective strategies of nutritional management and supplementation approaches for weight control in companion animals.

## Introduction

Obesity has become one of the most common medical disorders worldwide, both for humans and pets (1–3). The incidence of obesity in dogs has increased by up to 56% in the United States and is still sharply increasing, particularly among aged dogs (4). Obese dogs tend to be less active or social, which in turn aggravates obesity (5, 6). As one of the most common nutritional disorders, obesity is characterized by excess fat accumulation under the skin and abdomen in dogs. Excess body fat is a consequence of a prolonged imbalance between energy intake and expenditure (7). The obese condition is intimately correlated with decreased longevity and various complications, such as orthopedic disorders, glucose dysregulation, hypertriglyceridemia, cardiorespiratory dysfunction, liver dysfunction, immunological disorders, and carcinoma (8).

The current weight management strategies mainly include increasing energy expenditure through increased pet activity and reducing energy intake, either using drug therapy or a purpose-formulated weight-loss diet (9). Despite energy restriction by dietary therapy successfully leads to weight loss in canines, long-lasting treatment by multiple weight-loss diet may cause excessive protein loss that requires hospitalization monitoring (10). Routine diets just simply supplemented with higher protein or fiber proportions could effectively reduce body mass by providing adequate satiety and reducing energy intake but might induce malnutrition, especially in dogs with comorbidities (11–14). Another effective strategy is to explore effective drugs for canine obesity. Thus far, dirlotapide represents the unique approved first-in-class anti-obesity drug for dogs but would induces extensive adverse effects, including vomiting, loose stools, diarrhea, lethargy, anorexia, and serum transaminase elevations (15). Application of natural products holds inherent advantages for obesity and metabolic homeostasis. Among them, astaxanthin, a common natural antioxidant product, has been shown to ameliorate obesity, insulin resistance and hepatic steatosis in mice and dogs (16–19). Astaxanthin has been reported as a potent antioxidant and lipid profile regulator that provides health benefits to both humans and animals and is therefore commonly used in food (20–23). The role of astaxanthin has not been tested in commercial food products. The most popular and easy method for weight-loss therapy is a weight-loss program based on energy restriction in combination with dietary supplements for steady-state maintenance (3, 24).

In this study, we designed a formulated and balanced nutritional weight-loss diet with high protein, high fiber, and low-fat contents. The effects of this diet on canine obesity were fully evaluated in obese beagles *via* physical examination and transcriptional analysis. Our results showed that the formulated diet effectively reduced body weight and adipose tissue abundance in canines without toxicity.

## Materials and methods

### Study dogs

Healthy beagles aged 1 year old weighting 7.3–10.6 kg were used in the present study. After 2 weeks of acclimation, the beagles subjected to physical examination. All beagles were maintained in separate cages (length × width × height of 100 cm × 100 cm × 208 cm) in the animal facility on a 12-h light/dark cycle and 50–70% relative humidity with free access to water. During the experiment, food intake and physical activity were recorded daily by the researcher. The experiment was approved by the Biomedical Research Ethics Committee of Gannan Medical University, and all animal operations were in compliance with the operating guidelines of the Biomedical Ethics Committee of Gannan Medical University.

### Weight-loss regimen

The healthy beagles were randomly grouped into a normal chow group (NC group) and a high-fat diet group (HFD group) as previously reported (*n* = 15 dogs/group) (25). Briefly, the NC group dogs were fed a basal maintenance diet, and animals in the HFD group were treated by a customized diet. The compositions of the NC diets were as follows: total energy at 3615 kcal/kg (energy ratios: carbohydrate 59.2%; protein 27.1%, fat 13.7%; quality ratios: carbohydrate 53.5%, protein 24.5%; fat 5.5%, fiber 3.0%, ash 6.0%, mineral 1.8%). The total energy of HFD diet is 4832 kcal/kg (energy ratios: carbohydrate 26.9%, protein 19.8%, fat 53.3%; quality ratios: carbohydrate 32.5%; protein 24.0%, fat 28.6%, fiber 1.4%, ash 4.5%, mineral 1.7%). The two groups of beagles were continuously fed for 24 weeks, and physical examination, hematological examination, imaging examination and fat mass assay were performed to evaluate the degree of obesity. The obese dogs were screened and selected by conditional cutoff of the scoring scale for obesity (BCS ≥ 6). Then, the obese beagles were randomly divided into a weight-loss diet group and a control commercial diet group (*n* = 5 dogs/group). The compositions and nutrients of the weight-loss diet diets and control commercial diet were as follows: The total energy of control diet is 3249.4 kcal/kg (energy ratios: carbohydrate 43.27%, protein 23.33%, fat 33.4%; quality ratios: carbohydrate 51.73%, protein 18.95%; fat 12.06%, fiber 9.0%, ash 9.61%). The total energy of weight-loss diet is 3102.9 kcal/kg (energy ratios: carbohydrate 29.47%, protein 44.28%, fat 26.25%; quality ratios: carbohydrate 44.87%; protein 34.35%, fat 9.05%, fiber 15.11%, ash 7.25%). During diet feeding, dogs received routine physical examination, routine hematology measurement and biochemistry analysis. The control commercial diet was the full-price compound food for the experimental dogs, and the weight-loss diet for dogs was jointly developed by the Gannan Innovation and Translational Medicine Research Institute and Jiangxi Huichong Technology Co., Ltd.

### Physical examination

Beagles were weighed using the same electronic scales and a 9-point body condition score (BCS) evaluation, where BCS 1∼3 is considered underweight, BCS 4∼5 is lean, BCS 6∼7 is overweight or heavy and BCS 8∼9 is obese or severely obese (26). All assessments were made by two independent veterinarians. All beagles were determined to be healthy based on physical examination, routine hematology measurement, biochemistry analysis and urinalysis. Physical examination was performed biweekly, and the body weight curves were calculated.

### Blood sampling and serological analyses

Blood samples were collected by a needle from the hind limb veins of dogs. Routine hematology was performed by an easy-to-use veterinary hematology analyzer (VETSCAN HM5, 790-0000, Abaxis, Inc., Union City, CA, USA) using anticoagulated whole blood. For the plasma biochemistry analysis, plasma samples were obtained by separating the supernatant after centrifugation of whole blood for 10 min (2500 rpm, 4°C). Then, the plasma was analyzed by a HITACHI Automatic Analyzer (3100, HITACHI, Tokyo, Japan) using KHB serial biochemical diagnosis kits. Urinalysis tests in HFD beagles were performed by a urine chemistry analyzer (VETSCAN UA, 1500-0012, Abaxis, Inc., Union City, CA, USA) using urinalysis test strips (UA06794, Abaxis, Inc., Union City, CA, USA).

### Computed tomography and magnetic resonance imaging

Computed tomography and magnetic resonance imaging are common methods for evaluating abdominal adiposity as previous described (25). Total adipose tissue (TAT) volume, including visceral adipose tissue (VAT) and subcutaneous adipose tissue (SAT) volume, was calculated at CT (SOMATOM Definition, Siemens Medical Solution, Erlangen, Germany) and MRI (MAGNETOM Prisma 3.0T, Siemens Medical Systems, Erlangen, Germany). Images were captured when the canine was anesthetized. For MRI imaging, the abdominal fat distribution was shown clearly in Dixon fat-only MR images and fat fraction map was calculated by a workstation (Syngo MR Workplace, Siemens Healthcare, Erlangen, Germany). The region of interest (ROI) in the MRI study is the level of the T9-T13 thoracic vertebra. The CT images were used to assess the fat distribution of the total body. Thresholds were manually set from −150 to −50 Hounsfield units for TAT volume analysis. The assessment of TAT volume and total volume was calculated with 3D Slicer software (V.4.10.2, Brigham and Women’s Hospital, Harvard Medical School, Boston, MA, USA). The percentage of TAT volume was obtained by dividing the TAT volume by the abdominal volume.

### Canine palatability studies

For the canine palatability study, 10 beagles participated and were randomly assigned into a weight-loss diet group and a control diet group (*n* = 5 beagles/group). Dogs were offered each diet for 1 h each day for 3 days. At the end of the hour, the remaining food was weighed to determine the food intake ratio. The intake ratio determination test was further performed in pet dogs in clinic.

### Histopathological analysis

After feeding with a HFD or NC diet for 24 weeks, dogs were anesthetized, an incision was made along the dorsal side of the body, and subcutaneous adipose tissue was collected. The adipose tissue was immediately fixed with formaldehyde for 24–72 h and then dehydrated and embedded in paraffin. The paraffin blocks were cut into slices using a rotary microtome. The paraffin-embedded sections were stained using hematoxylin and eosin (H&E). Histological images of tissue sections were obtained by a light microscope (Olympus, Tokyo, Japan). Similar operations were performed after the weight-loss diet feeding for 8 weeks.

### RNA extraction and real-time PCR

Total RNA from subcutaneous and visceral adipose tissue was obtained by TRIzol reagent (T9424; Sigma–Aldrich, St. Louis, MO, USA) according to the manufacturer’s protocol, and then mRNA was transcribed to cDNA. Gene expression was quantified on a real-time PCR System (LightCycler 480 Instrument II, Roche Diagnostics Inc., Basel, BS, Switzerland). The relative gene expression levels of the target genes were normalized to those of the housekeeping gene *Actin*. The primer sequences were as follows: *Pparg*, F: 5′-GCGACTCCTTTGCTGATACAC-3′, R: 5′-CGGAACTGACACCCCTGAAA-3′. *Cd36*, F: 5′-CCAGACC TCTGGAAACCACC-3′, R: 5′-CGGTCACAGCCCATTCTC TT-3′. *Elovl5*, F: 5′-CAAGGCATTACTAGGCCCCC-3′, R: 5′-ATTCCCCGGCAAGAGAATGG-3′. *Acaa2*, F: 5′-TC GACAGCATCATCGTAGGC-3′, R: 5′-CAACGAAAGCTGGG ACCTCT-3′ *Hacd2*, F: 5′-CCTCATCAAATGGGCCAGGT-3′, R: 5′-CTGATGGAGTACAGGCCAGC-3′. *Il6*, F: 5′-CTCCTG ACCCAACCACAGAC-3′, R: 5′-GGAAATCCTCCAGACTC CGC-3′.

### RNA-seq and gene set enrichment analysis

We analyzed the transcriptomes of both the subcutaneous adipose tissue and visceral adipose tissue obtained from beagles. Briefly, total RNA was extracted, and then the RNA quality was examined by agarose electrophoresis and an RNA 6000 Nano Kit (#5067-1511; Agilent, Palo Alto, CA, USA). For RNA-seq, cDNA libraries were constructed using an MGIEasy RNA Library Prep Kit (1000006384; MGI, Shenzhen, China) according to the manufacturer’s instructions. Then, gene expression profiling was performed in a BGISEQ-2000 instrument (MGI, Shenzhen, China) by sequencing as previously reported (27). Briefly, following mRNA enrichment, samples were incubated with fragmentation buffer and then reverse-transcribed into cDNA. After repair and A tailing, the double strand cDNA products were ligated with adapter, and subjected to PCR amplification. After cleanup of PCR products using DNA clean beads, quality control of purified PCR product was carried out by Agilent DNA 1000 kit. PCR products were subjected to multiple sample pooling, cyclization and digestion to obtain the resulting libraries before sequencing. GSEA was implemented on the Java GSEA version 3.0 platform with the “Signal2Noise” metric to generate a ranked list and a “gene set” permutation type. Gene sets with FDR values less than 0.25 and nominal *P*-values less than 0.05 were considered statistically significant.

### Statistical analysis

SPSS 26.0 software was used to analyze the datasets. Student’s *t*-test was used to compare the significance of the difference between 2 groups that met a normal distribution, and the Mann–Whitney *U* non-parametric test was applied to compare two independent samples from skewed distributions. A *P*-value less than 0.05 was considered statistically significant.

## Results

### High-fat diet feeding successfully induced obesity in beagles

**FIGURE 1:**
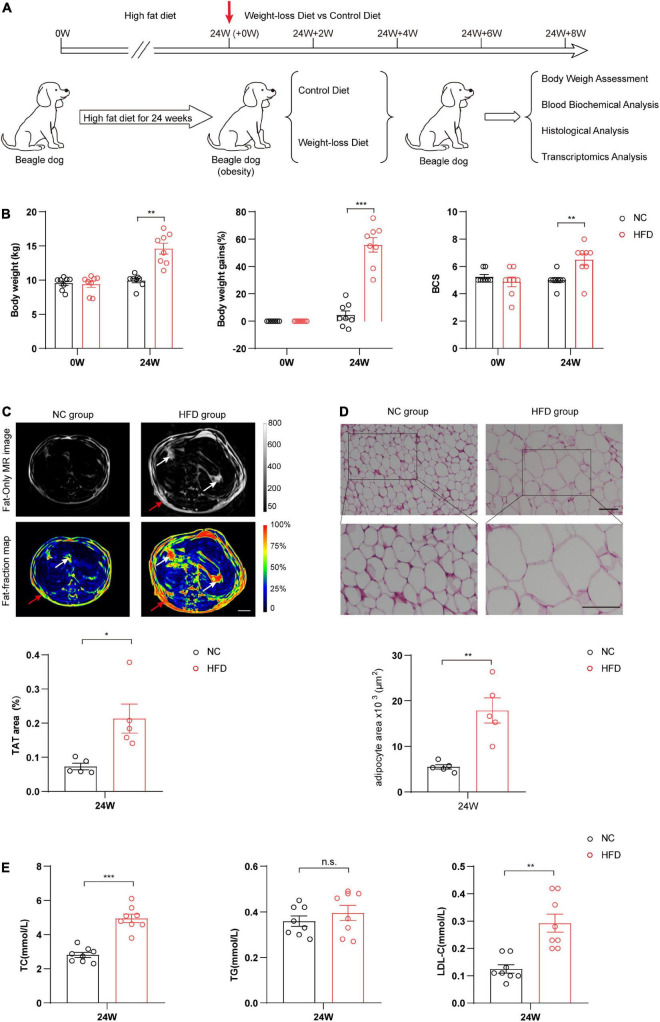
High-fat diet (HFD) successfully induced obesity in beagle dogs. **(A)** Schematic diagram of the experimental design process. Beagles were randomly divided into two groups, and then fed a HFD or a normal chow diet (NC) for 24 weeks. The obese dogs were evaluated by physical examination and laboratory and imaging examinations. Obese dogs were selected and randomly assigned to be fed with a weight-loss diet or control diet for an additional 8 weeks. **(B)** Analysis of body weight, body weight gain, and body condition scoring (BCS) in the HFD and NC groups at 0 and 24 weeks. *n* = 8 beagles/group. **(C)** Cross-sectional nuclear magnetic resonance imaging (MRI) analysis of the fat distribution in the HFD and NC groups at 0 and 24 weeks. Total adipose tissue (TAT) quantification was calculated based on the distributions of subcutaneous adipose tissue (SAT) (red arrow) and visceral adipose tissue (VAT) (white arrow). *n* = 5 beagles/group. Scale bar, 5 cm. **(D)** Representative images of H&E staining and comparison of morphological changes of adipose tissue between two groups of beagles after 24 weeks. *n* = 5 beagles/group. Scale bar, 100 μm. **(E)** The levels of TG, TC, and LDL in serum reflecting the blood lipids. *n* = 8 beagles/group. The data of body weight and body weight gains in panel **(B)** and the data in panels **(C–E)** are presented as the means ± SEMs and analyzed by Student’s two-tailed *t*-test. The data of BCS in panel **(B)** are presented as the means ± SEMs and analyzed by the Mann–Whitney *U* non-parametric test. *P < 0.05; **P < 0.01; ***P < 0.001. TG, triglyceride; TC, total cholesterol; LDL-C, low-density lipoprotein cholesterol.

To systematically investigate the effects of a weight-loss diet in obese dogs, we first constructed the obese model in beagles *via* 24-week continuous HFD feeding. The obese dogs were selected by the conditional cutoff of the scoring scale for obesity (BCS ≥ 6), and ten beagles were used to evaluate the weight loss effects of the formulated weight-loss diet (Figure 1A). As shown in Figure 1B, compared with the NC group, the 24-week HFD significantly increased body weight (*P* < 0.01), body weight gain (*P* < 0.001), and BCS in beagles (*P* < 0.01). Cross-sectional nuclear magnetic resonance imaging (MRI) analysis showed that HFD markedly increased the fat mass of beagles, especially the fat distribution of the visceral adipose tissue (VAT) and subcutaneous adipose tissue (SAT) (*P* < 0.05) (Figure 1C). H&E staining of adipose tissues showed that the volumes of adipocytes were markedly increased in the HFD group, suggesting significant adipocyte hypertrophy during canine obesity (*P* < 0.05) (Figure 1D). Hematological analysis showed that the HFD also obviously increased the plasma lipid content, such as total cholesterol (TC) (*P* < 0.001) and low-density lipoprotein (LDL) (*P* < 0.01), and slightly increased the plasma triglyceride (TG) content (Figure 1E). Taken together, these data indicate that a canine obesity model was successfully induced by HFD feeding.

### Composition and energy analysis of the weight-loss diet

**FIGURE 2:**
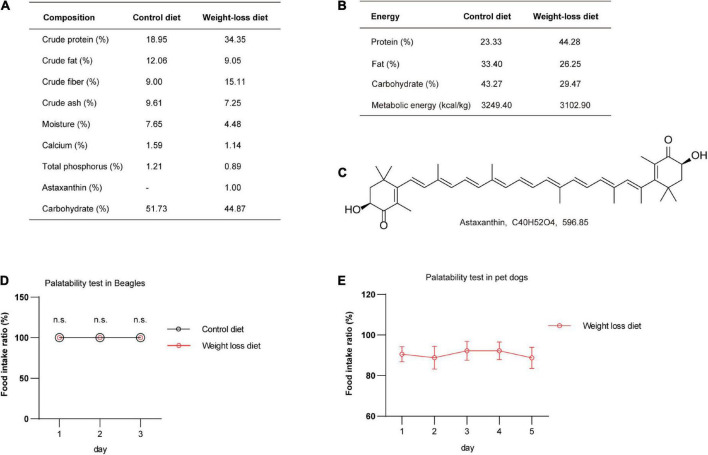
The nutritional composition of the weight-loss diet and food palatability evaluation. **(A,B)** The nutritional composition **(A)** and energy supply ratio **(B)** of the weight-loss diet and control diet. **(C)** The chemical structure of the astaxanthin addictive ingredient. **(D)** The palatability testing analysis in beagles fed a weight-loss diet and control diet in the indicated days. *n* = 5 beagles/group. **(E)** The palatability testing analysis in pet dogs fed a weight-loss diet in the indicated days. *n* = 9 pet dogs. The data in panels **(D,E)** are presented as the means ± SEMs and were analyzed by Student’s two-tailed *t*-test. n.s., not significant.

Numerous reports have indicated that formulated diets containing high contents of protein and fiber have significant satiating and weight reduction effects (12, 14, 28). Natural plant products are often added to diets as flavoring agents or functional active substances. Astaxanthin has been reported having anti-obese and antidiabetic effects in mice (16, 29). Here, we added astaxanthin as a food additive to the weight-loss diet. The dietary chemical composition and energy supplies of the formulated weight-loss diet and control diet were systematically analyzed. The detailed information is presented in Figures 2A,B. The molecular structure of astaxanthin is shown in Figure 2C. To investigate the taste and palatability of the formulated weight-loss diet, we first performed a palatability test in 10 healthy beagles. The results showed that food intake ratios were 100% in both groups, and there was no significant difference in feed intake in 3-day duration in dogs feeding with the weight-loss diet and control diet (Figure 2D). To further investigate palatability, pet dogs were recruited and assayed for palatability for 5 days, and it was observed that weight-loss diet administration showed good palatability in pet dogs (Figure 2E).

### The formulated weight-loss diet significantly alleviates obesity in beagles

**FIGURE 3:**
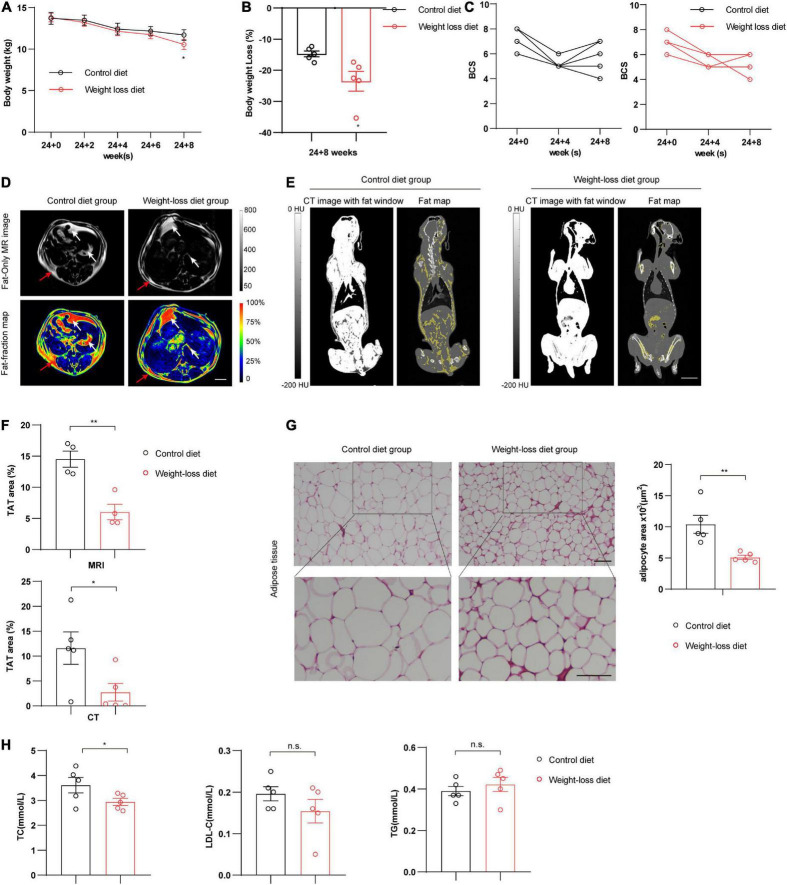
Body weight and fat mass evaluation in beagles. **(A–C)** Analysis of body weight **(A)**, body weight loss **(B)**, and body condition scoring (BCS) **(C)** in beagles fed a weight-loss diet and a control diet at the indicated time points. *n* = 5 beagles/group. **(D)** Representative cross-sectional MRI showed the fat distribution in beagles at 8 weeks in the indicated groups. *n* = 4 beagles/group. Scale bar, 2 cm. Quantifications of TAT% based on MRI are shown on the right. *n* = 4 beagles/group. **(E)** Representative images of computed tomography (CT) showed the fat mass distribution in coronal sections in beagles fed a weight-loss diet and control diet at 8 weeks. Grayscale and pseudocolor images represent the proportion of the total adipose tissue (TAT%). *n* = 5 beagles/group. Scale bar, 5 cm. HU, Hounsfield unit. **(F)** Quantification of TAT% based on MRI and CT. TAT quantification was calculated based on the distributions of subcutaneous adipose tissue (SAT) (red arrow) and visceral adipose tissue (VAT) (white arrow). *n* = 4∼5 beagles/group. **(G)** Representative images of H&E staining in adipose tissues of control diet and weight-loss diet beagles feeding for 8 weeks. *n* = 5 beagles/group. Scale bar, 100 μm. **(H)** Serum levels of total cholesterol (TC), triglyceride (TG), and low-density lipoprotein (LDL) in beagles fed a weight-loss diet and a control diet at 8 weeks. *n* = 5 beagles/group. All the data are presented as the means ± SEMs and were analyzed by Student’s two-tailed *t*-test. *P < 0.05; **P < 0.01. n.s., not significant.

Obesity is defined as an accumulation of excessive amounts of adipose tissue in the body. Potential techniques for evaluating obesity include total body weight or body water measurement, plasma chemical analysis, densitometry, absorptiometry, ultrasonography, and advanced comprehensive imaging techniques (such as CT and MRI) (10). Compared to the control diet group, the weight-loss diet group showed obvious reductions in body weight and body weight loss at the 8-week time point after feeding (*P* < 0.05) (Figures 3A,B). We also observed a declining trend in BCS scores (Figure 3C). We next analyzed the fat mass by advanced imaging techniques. The distribution of adipose tissues in beagles was clearly visualized by MRI (Figure 3D) and CT (Figure 3E) after feeding a weight-loss diet and control diet for 8 weeks. Compared to that in the control diet group, total adipose tissue (TAT) was significantly decreased in the weight-loss diet group as assessed by MRI (*P* < 0.01) and CT analysis (*P* < 0.05) (Figure 3F). Further pathological analysis of adipose tissue showed that the subcutaneous adipocyte size was markedly reduced in the weight-loss group (*P* < 0.01) (Figure 3G). Plasma biochemistry examination data showed lower plasma lipids in the weight-loss group, especially the TC content (*P* < 0.05) (Figure 3H). Taken together, these data indicate that the formulated weight-loss diet protects against obesity in beagles, exhibiting profound beneficial effects on canine obesity.

### The formulated obesity control diet improved the whole metabolic panels of subcutaneous adipose tissue in beagles

**FIGURE 4:**
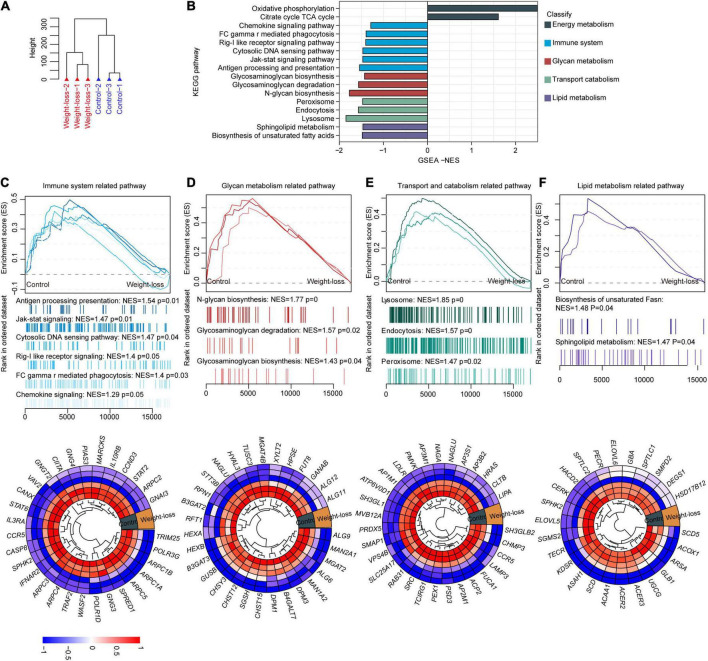
The metabolic signature of subcutaneous adipose tissue in a weight-loss diet in beagles. **(A)** Total RNA was extracted from subcutaneous adipose tissues of control diet and weight-loss diet beagles after 8 weeks of feeding followed by RNA-seq analysis. PCA Cluster showed the distribution of samples from beagles fed a weight-loss diet control diet according to gene expression patterns in RNA-seq. *n* = 3 beagles/group. **(B)** Gene set enrichment analysis (GSEA) was carried out between the control diet and weight-loss diet beagles. GSEA showing the enriched pathways related to lipid metabolism based on the RNA-seq dataset in control diet and weight-loss diet beagles after 8 weeks of feeding. **(C)** Representative GSEA enrichment score curves of immune system-related pathways. Genes on the far left correlated the most with control diet samples, and genes on the far right correlated the most with weight-loss diet samples. A representative heatmap of the leading genes is shown below the curve. **(D)** Representative GSEA enrichment score curves and heatmap of the glycan metabolism-related pathways and corresponding leading genes. **(E)** Representative GSEA enrichment score curves and heatmap of the transport catabolism-related pathways and corresponding leading genes. **(F)** Representative GSEA enrichment score curves and heatmap of the lipid metabolism-related pathways and corresponding leading genes.

Adipose tissue, largely regulates body energy homeostasis and maintenance of body mass (30). To fully evaluate the influences of the developed food formula on whole homeostasis in an unbiased manner, we further performed RNA-seq assays on subcutaneous and visceral adipose tissues from beagles in the control and obesity control diet groups after 8 weeks of feeding. Unsupervised hierarchical clustering analysis of subcutaneous adipose tissues separated the samples into two independent clusters, indicating the robust influences of the designed diet on adipose tissues (Figure 4A). Gene set enrichment analysis (GSEA) clearly showed that the downregulated genes induced by obesity control diet were highly enriched in pro-obesity-related pathways, mainly including the chemokine signaling pathway, Rig-I-like receptor signaling pathway, cytosolic DNA sensing pathway, Jak-stat signaling pathway, glycosaminoglycan biosynthesis, glycosaminoglycan degradation, peroxisome, lysosome, sphingolipid metabolism and biosynthesis of unsaturated fatty acids, while oxidative phosphorylation and TCA cycle pathways were significantly upregulated by the developed diet feeding (Figure 4B). Specifically, pathways related to lipid metabolism, immune system, glycan metabolism, and transport catabolism were downregulated in the weight-loss group (Figures 4C–F). Furthermore, the leading genes of those indicated pathways were also changed accordingly (Figures 4C–F). Taken together, these data indicate that the weight-loss diet improves obesity by downregulating the pro-obesity-related metabolic signature in adipose tissues.

### The weight-loss diet improved metabolic signature of visceral adipose tissue in beagles

**FIGURE 5:**
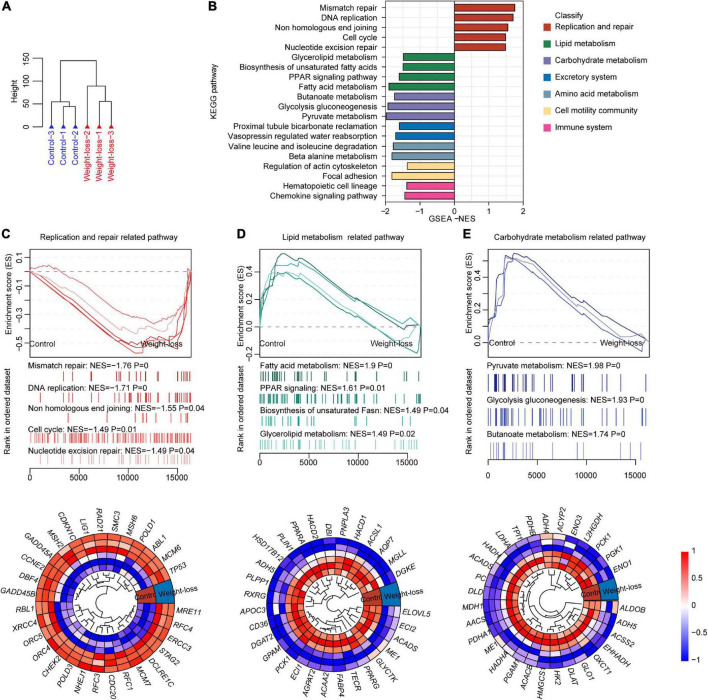
The metabolic signature of visceral adipose tissue in a weight-loss diet in beagles. **(A)** Total RNA was extracted from visceral adipose tissues of control diet and weight-loss diet beagles after 8 weeks of feeding and then subjected to RNA-seq. Cluster images show the distribution of samples from control diet and weight-loss diet beagles according to gene expression patterns in RNA-seq. *n* = 3 beagles/group. **(B)** Gene set enrichment analysis (GSEA) was carried out between the control diet and weight-loss diet beagles. GSEA showing the enriched pathways related to lipid metabolism based on the RNA-seq dataset in control diet and weight-loss diet beagles after 8 weeks of feeding. **(C)** Representative GSEA enrichment score curves of the replication and repair-related pathways. Genes on the far left correlated the most with control diet samples, and genes on the far right correlated the most with weight-loss diet samples. A representative heatmap of the leading genes is shown below the curve. **(D)** Representative GSEA enrichment score curves and heatmap of the lipid metabolism-related pathways and corresponding leading genes. **(E)** Representative GSEA enrichment score curves and heatmap of the carbohydrate metabolism-related pathways and corresponding leading genes.

We further investigated the effects of a weight-loss diet on visceral adipose tissue *via* RNA-seq analysis. Unsupervised hierarchical clustering analysis indicated the robust influences of the designed diet on adipose tissues (Figure 5A). The weight-loss diet feeding led to significantly inactivation of lipid metabolism, carbohydrate metabolism, excretory system, amino acid metabolism, cell motility community, and immune system, while replication and repair pathways were significantly upregulated by the developed diet (Figure 5B). Furthermore, the pathways related to DNA replication, non-homologous end joining, cell cycle, and nucleotide excision repair were upregulated in the weight-loss diet group (Figure 5C), and pathways related to fatty acid metabolism, PPAR signaling, biosynthesis of unsaturated Fatty acid, glycerolipid metabolism, pyruvate metabolism, glycolysis, gluconeogenesis, and butanoate metabolism were downregulated in the weight-loss group (Figures 5D,E). Altogether, the weight-loss diet exhibited profound effects on energy metabolic homeostasis that is essential for the maintenance of body mass.

### The weight-loss diet effectively ameliorated the whole metabolic and immune profiles in adipose tissues

**FIGURE 6:**
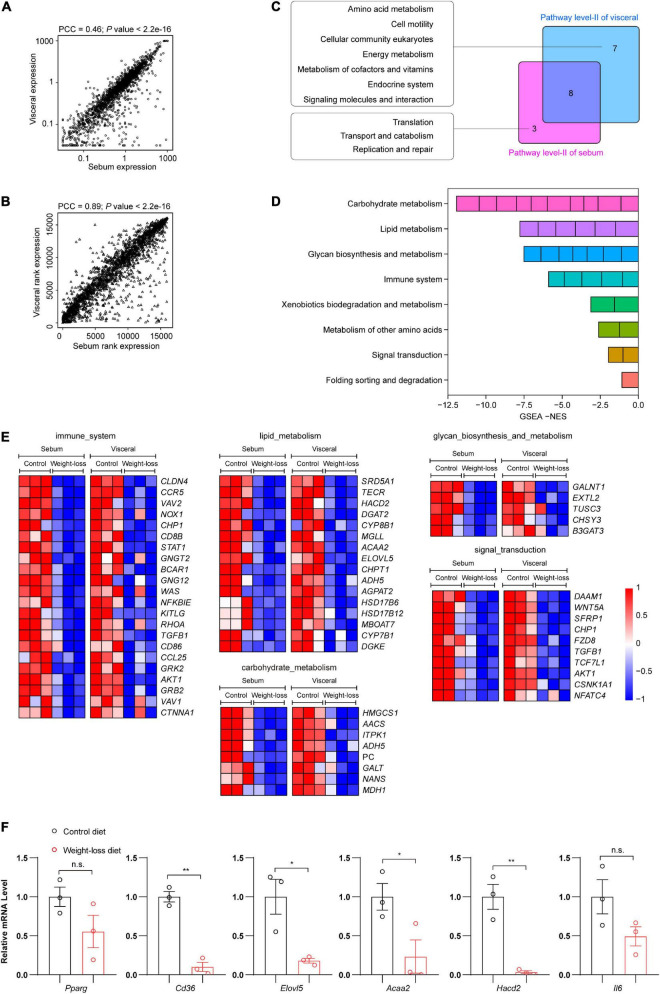
The weight-loss diet effectively ameliorated the whole metabolic and immune profiles in adipose tissues. **(A)** Correlation analysis of total gene expression in RNA-seq between subcutaneous and visceral adipose tissues. **(B)** Correlation analysis of gene expression rank in RNA-seq between subcutaneous and visceral adipose tissues. **(C)** A venn diagram showing the numbers of unique and shared regulated molecular events between subcutaneous and visceral adipose tissues. **(D)** Detailed information on the shared regulated molecular events from the RNA-seq analysis. **(E)** Heatmaps of the core events regulated by the weight-loss diet in the subcutaneous and visceral adipose tissues. **(F)** Representative gene expression in subcutaneous adipose tissues by qPCR analysis. The data in panel **(F)** are presented as the means ± SEMs and were analyzed by Student’s two-tailed *t*-test. *P < 0.05, **P < 0.01; n.s., not significant.

To further decipher the similarity and specificity of the transcriptional profiles in subcutaneous and visceral adipose tissues regulated by the weight-loss diet, we performed a correlation analysis of RNA-seq data between the sebum and visceral fat samples. The Pearson correlation coefficient (PCC) was used to analyze the correlations of total gene expression (Figure 6A) and gene expression rank (Figure 6B) in the indicated samples. The transcriptome signature between subcutaneous and visceral adipose tissues was highly significant and connectively correlated. Thus, we next analyzed the common transcriptome signature between sebum and visceral fat and found that they shared 8 common molecular events, including immune system, lipid metabolism, carbohydrate metabolism, glycan biosynthesis and metabolism, and signal transduction. Among them, the immune system, lipid metabolism and carbohydrate metabolism ranked as the top three most significant ones (Figures 6C,D). Compared to the control diet, the weight-loss diet exhibited profound anti-obese effects by regulating the common core genes in sebum and visceral fat (Figure 6E). We also confirmed the RNA-seq data by further gene expression validation through qPCR analysis. Consistently, compared to the control diet group, genes related to lipid metabolism and inflammation were markedly downregulated in the weight-loss diet group (Figure 6F).

### The formulated weight-loss diet showed high safety during the weight-loss program in beagles

**FIGURE 7:**
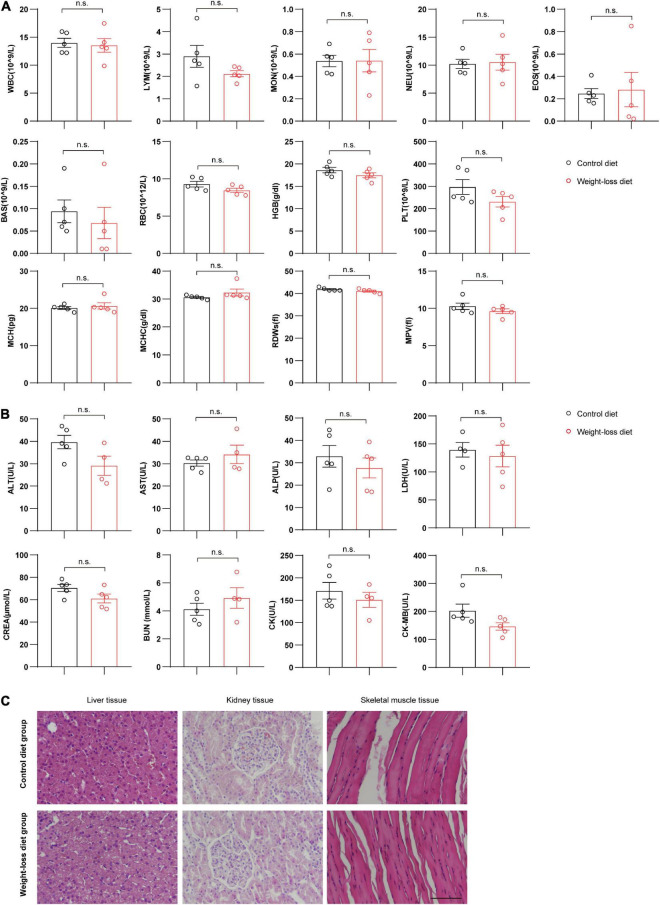
Evaluation of the adverse effects in beagles fed a weight-loss diet. **(A)** Whole blood parameter analysis of beagles fed a weight-loss diet and a control diet. *n* = 5 beagles/group. **(B)** Blood plasma biochemical evaluation of beagles in the indicated groups. *n* = 4∼5 beagles/group. **(C)** H&E staining evaluation of the morphological changes in liver tissues, kidney tissues and skeletal muscle tissues in beagles fed the indicated diet for 8 weeks. *n* = 5 beagles/group. Scale bar, 100 μm. The data of RDWs in panel **(A)** are presented as the means ± SEMs and analyzed by the Mann–Whitney *U* non-parametric test and the other data in panels **(A,B)** are presented as the means ± SEMs and analyzed by Student’s two-tailed *t*-test. n.s., not significant; WBC, white blood cell; LYM, lymphocytes; MON, monocytes; NEU, neutrophils; EOS, eosinophils; BAS, basophils; RBC, red blood cell; HGB, hemoglobin; PLT, platelets; MCH, mean corpuscular hemoglobin; RDW, red cell distribution width; MPV, mean platelet volume; MCHC, mean corpuscular hemoglobin concentration; ALT, alanine aminotransferase; AST, aspartate aminotransferase; ALP, alkaline phosphatase; LDH, lactate dehydrogenase; CREA, creatinine; BUN, blood urea nitrogen; CK, creatine kinase; CK-MB, creatine kinase MB.

To fully investigate the safety of the formulated weight-loss diet, we systematically evaluated the hematological and biochemical parameters after 8 weeks of diet feeding. The whole blood program of red blood cells, white blood cells and platelets were measured and analyzed. No significant differences were observed for any hematological parameters between the two tested groups (Figure 7A). The plasma biochemical parameters also showed that the plasma levels of alanine aminotransferase (ALT), aspartate aminotransferase (AST), alkaline phosphatase (ALP), lactate dehydrogenase (LDH), creatinine (CREA), blood urea nitrogen (BUN), creatine kinase (CK), and creatine kinase MB (CK-MB) were not obviously different between the weight-loss diet and control diet (Figure 7B). Further pathological staining of liver, kidney and skeletal muscle tissues also showed no adverse effects in either the weight-loss diet group or the control diet group (Figure 7C). These data indicate that the formulated diet has satisfactory anti-obesity effects with well palatability and safety.

## Discussion

Obesity occurs when dogs are in energy imbalance for an extended period of time due to either excessive dietary intake or inadequate energy utilization. It can be defined as a condition of excessive energy storage in the form of adipose tissue to a degree leading to adverse effects on health and longevity (7). In this study, we designed a formulated weight-loss diet and investigated the ability of this food to induce modest weight reduction in overweight beagle dogs. The weight-loss diet markedly decreased the body weight of obese dogs and reduced the volume of subcutaneous and abdominal fat mass. Transcriptional analysis of adipose tissues indicated that energy metabolism-related pathways were activated by 8-week weight-loss diet feeding, while pathways or genes related to pro-obesity, such as lipid synthesis, immune system, glycan metabolism, and transport catabolism, were markedly downregulated.

In recent decades, the incidence of overweight or obesity worldwide has increased significantly in dogs. The detailed mechanism of obesity in dogs has rarely been investigated, and most reports propose that obesity is a nutritional disorder. Thus, the most common method is the restriction of calories. An abundance of evidence suggests that providing balanced and commercial diets with higher protein and fiber proportions effectively provides adequate satiety and prevents malnutrition during the weight-loss program (14). Obesity is also a chronic metabolic disease, and adipose tissue homeostasis plays pivotal roles in the pathogenesis of obesity. Previous reports analyzed the subcutaneous and gonadal adipose tissue *via* microarray and indicated that genes associated with adipogenesis, energy metabolism, oxidation reduction, immune response, and extracellular matrix were significantly regulated in obese dogs (31, 32). These global changes in metabolic profile of adipose tissues reported previously are consistent with our investigations into subcutaneous adipose tissues of obese beagles fed a weight-loss diet by RNA-seq assay.

Although only a short period was monitored and evaluated, most dogs lost weight successfully with well safety under hemostatic condition. Furthermore, weight reduction occurred with more modest energy restriction than previous weight reduction studies (33). Astaxanthin prevents obesity-associated metabolic disturbances and inflammation, increases mitochondrial oxidative phosphorylation, and enhances exercise-induced fatty acid metabolism (18, 21). The effects of astaxanthin on obesity have been widely reported in mice (18, 19, 21, 34) but rarely reported in dogs (17). Numerous studies have suggested that a high-protein/high-fiber diet exhibits weight-loss effects on obese dogs (11, 12, 14). In our research, we designed a formulated high-protein and fiber diet supplemented with astaxanthin and found that this diet alleviated high-fat diet-induced obesity in beagle dogs. Although commercially available astaxanthin-supplemented dog food is on the market (CN112515049A), it is often used to enhance the immune response, beautify hair, and delay aging in dogs. At present, there is no prescription food supplemented with astaxanthin used for the prevention and treatment of canine obesity and related metabolic syndrome on the market. In this study, we focus on the function and safety of the designed weight-loss diet. The formulation and composition of the weight-loss diet and functional research on the weight-loss diet in canine obesity are also completely innovative. Here we observed a significant reduction in weight loss in the weight-loss diet-fed dogs and also found that a weight-loss diet significantly increased energy metabolism-related pathways and decreased lipid synthesis-related pathways. We suppose that the weight-loss effect was the synergy of the high-protein high-fiber diet and the high concentration of the plant additive astaxanthin. A limitation of the study was the fact that we only tested the effects of a weight-loss diet in experimental HFD-induced obese beagles, and the effects of this diet were not further validated in pet dogs. Further clinical investigations will be performed in a large range of clinical animals and the effects of the formulated weight-loss diet will be widely validated.

## Data availability statement

The RNA-seq data presented in this study are deposited in the NCBI SRA repository with accession number: PRJNA884431. The names of the repository/repositories and accession number(s) can be found at: https://www.ncbi.nlm.nih.gov/sra.

## Ethics statement

This animal study was reviewed and approved by Biomedical Research Ethics Committee of Gannan Medical University. Written informed consent was obtained from the owners for the participation of their animals in this study.

## Author contributions

JX and YL designed and performed the experiments, analyzed the data, and wrote the manuscript. TZ, WS, SW, XC, JW, YC, and QW performed animal, pathological, and imageological experiments, omic analysis, and analyzed the data. MW and MH provided the methodology of the diet and analyzed data. XY, MD, ZQ, YD, and XZ helped to design the study and provided valuable suggestions. YH and HL provided the study concept and design, edited the manuscript, and supervised the study. All authors approved the final version of the manuscript.

## References

[1] Chooi YC Ding C Magkos F. The epidemiology of obesity. *Metabolism.* (2019) 92:6–10. 10.1016/j.metabol.2018.09.005 30253139

[2] Bluher M. Obesity: global epidemiology and pathogenesis. *Nat Rev Endocrinol.* (2019) 15:288–98. 10.1038/s41574-019-0176-8 30814686

[3] Shepherd M. Canine and feline obesity management. *Vet Clin North Am Small Anim Pract.* (2021) 51:653–67. 10.1016/j.cvsm.2021.01.005 33653534

[4] Loftus JP Wakshlag JJ. Canine and feline obesity: a review of pathophysiology, epidemiology, and clinical management. *Vet Med (Auckl).* (2015) 6:49–60. 10.2147/VMRR.S40868 30101096 PMC6067794

[5] Salt C Morris PJ Wilson D Lund EM German AJ. Association between life span and body condition in neutered client-owned dogs. *J Vet Intern Med.* (2019) 33:89–99. 10.1111/jvim.15367 30548336 PMC6335446

[6] Morrison R Penpraze V Beber A Reilly JJ Yam PS. Associations between obesity and physical activity in dogs: a preliminary investigation. *J Small Anim Pract.* (2013) 54:570–4. 10.1111/jsap.12142 24117778

[7] Courcier EA Thomson RM Mellor DJ Yam PS. An epidemiological study of environmental factors associated with canine obesity. *J Small Anim Pract.* (2010) 51:362–7. 10.1111/j.1748-5827.2010.00933.x 20402841

[8] Osto M Lutz TA. Translational value of animal models of obesity-Focus on dogs and cats. *Eur J Pharmacol.* (2015) 759:240–52. 10.1016/j.ejphar.2015.03.036 25814247

[9] Brooks D Churchill J Fein K Linder D Michel KE Tudor K 2014 AAHA weight management guidelines for dogs and cats. *J Am Anim Hosp Assoc.* (2014) 50:1–11. 10.5326/JAAHA-MS-6331 24216501

[10] German AJ. The growing problem of obesity in dogs and cats. *J Nutr.* (2006) 136:1940S–6S. 10.1093/jn/136.7.1940S 16772464

[11] Phungviwatnikul T Lee AH Belchik SE Suchodolski JS Swanson KS. Weight loss and high-protein, high-fiber diet consumption impact blood metabolite profiles, body composition, voluntary physical activity, fecal microbiota, and fecal metabolites of adult dogs. *J Anim Sci.* (2022) 100:skab379. 10.1093/jas/skab379 34967874 PMC8846339

[12] Bermudez Sanchez S Pilla R Sarawichitr B Gramenzi A Marsilio F Steiner JM Untargeted fecal metabolome analysis in obese dogs after weight loss achieved by feeding a high-fiber-high-protein diet. *Metabolomics.* (2021) 17:66. 10.1007/s11306-021-01815-1 34228201 PMC8260550

[13] Ko GJ Rhee CM Kalantar-Zadeh K Joshi S. The effects of high-protein diets on kidney health and longevity. *J Am Soc Nephrol.* (2020) 31:1667–79. 10.1681/ASN.2020010028 32669325 PMC7460905

[14] Weber M Bissot T Servet E Sergheraert R Biourge V German AJ. A high-protein, high-fiber diet designed for weight loss improves satiety in dogs. *J Vet Intern Med.* (2007) 21:1203–8. 10.1892/07-016.1 18196727

[15] Klonoff DC. Dirlotapide, a U.S. food and drug administration-approved first-in-class obesity drug for dogs-will humans be next? *J Diabetes Sci Technol.* (2007) 1:314–6. 10.1177/193229680700100301 19885086 PMC2769592

[16] Ikeuchi M Koyama T Takahashi J Yazawa K. Effects of astaxanthin in obese mice fed a high-fat diet. *Biosci Biotechnol Biochem.* (2007) 71:893–9. 10.1271/bbb.60521 17420580

[17] Murai T Kawasumi K Tominaga K Okada Y Kobayashi M Arai T. Effects of astaxanthin supplementation in healthy and obese dogs. *Vet Med (Auckl).* (2019) 10:29–35. 10.2147/VMRR.S186202 30859086 PMC6385744

[18] Nishida Y Nawaz A Kado T Takikawa A Igarashi Y Onogi Y Astaxanthin stimulates mitochondrial biogenesis in insulin resistant muscle via activation of AMPK pathway. *J Cachexia Sarcopenia Muscle.* (2020) 11:241–58. 10.1002/jcsm.12530 32003547 PMC7015247

[19] Wang M Ma H Guan S Luo T Zhao C Cai G Astaxanthin from *Haematococcus pluvialis* alleviates obesity by modulating lipid metabolism and gut microbiota in mice fed a high-fat diet. *Food Funct.* (2021) 12:9719–38. 10.1039/d1fo01495a 34664590

[20] Walker LA Wang T Xin H Dolde D. Supplementation of laying-hen feed with palm tocos and algae astaxanthin for egg yolk nutrient enrichment. *J Agric Food Chem.* (2012) 60:1989–99. 10.1021/jf204763f 22276647

[21] Wu L Lyu Y Srinivasagan R Wu J Ojo B Tang M Astaxanthin-shifted gut microbiota is associated with inflammation and metabolic homeostasis in mice. *J Nutr.* (2020) 150:2687–98. 10.1093/jn/nxaa222 32810865 PMC8023541

[22] Gateau H Solymosi K Marchand J Schoefs B. Carotenoids of microalgae used in food industry and medicine. *Mini Rev Med Chem.* (2017) 17:1140–72. 10.2174/1389557516666160808123841 27515712

[23] Janiszewska-Turak E. Carotenoids microencapsulation by spray drying method and supercritical micronization. *Food Res Int.* (2017) 99:891–901. 10.1016/j.foodres.2017.02.001 28847426

[24] Olivindo RFG Zafalon RVA Teixeira FA Vendramini THA Pedrinelli V Brunetto MA. Evaluation of the nutrients supplied by veterinary diets commercialized in Brazil for obese dogs undergoing a weight loss program. *J Anim Physiol Anim Nutr (Berl).* (2022) 106:355–67. 10.1111/jpn.13689 35112401

[25] Qu W Chen Z Hu X Zou T Huang Y Zhang Y Profound perturbation in the metabolome of a canine obesity and metabolic disorder model. *Front Endocrinol.* (2022) 13:849060. 10.3389/fendo.2022.849060 35620391 PMC9128610

[26] German AJ Holden SL Moxham GL Holmes KL Hackett RM Rawlings JM. A simple, reliable tool for owners to assess the body condition of their dog or cat. *J Nutr.* (2006) 136:2031S–3S. 10.1093/jn/136.7.2031S 16772488

[27] Hu Y He W Huang Y Xiang H Guo J Che Y FASN-suppressor screening identifies SNX8 as a novel therapeutic target for NAFLD. *Hepatology.* (2021) 74:2508–25. 10.1002/hep.32045 34231239

[28] Keller E Sagols E Flanagan J Biourge V German AJ. Use of reduced-energy content maintenance diets for modest weight reduction in overweight cats and dogs. *Res Vet Sci.* (2020) 131:194–205. 10.1016/j.rvsc.2020.04.019 32388022

[29] Bhuvaneswari S Arunkumar E Viswanathan P Anuradha CV. Astaxanthin restricts weight gain, promotes insulin sensitivity and curtails fatty liver disease in mice fed a obesity-promoting diet. *Process Biochem.* (2010) 45:1406–14. 10.1016/j.procbio.2010.05.016

[30] Kuryszko J Slawuta P Sapikowski G. Secretory function of adipose tissue. *Pol J Vet Sci.* (2016) 19:441–6. 10.1515/pjvs-2016-0056 27487522

[31] Grant RW Vester Boler BM Ridge TK Graves TK Swanson KS. Adipose tissue transcriptome changes during obesity development in female dogs. *Physiol Genomics.* (2011) 43:295–307. 10.1152/physiolgenomics.00190.2010 21224421

[32] Grant RW Vester Boler BM Ridge TK Graves TK Swanson KS. Subcutaneous and gonadal adipose tissue transcriptome differences in lean and obese female dogs. *Anim Genet.* (2013) 44:728–35. 10.1111/age.12060 23713485

[33] Gaylord L Remillard R Saker K. Risk of nutritional deficiencies for dogs on a weight loss plan. *J Small Anim Pract.* (2018) 59:695–703. 10.1111/jsap.12913 30117159

[34] Yang Y Pham TX Wegner CJ Kim B Ku CS Park YK Astaxanthin lowers plasma TAG concentrations and increases hepatic antioxidant gene expression in diet-induced obesity mice. *Br J Nutr.* (2014) 112:1797–804. 10.1017/S0007114514002554 25328157

